# Accelerated innovation through repurposing: exaptation of design and manufacturing in response to COVID‐19

**DOI:** 10.1111/radm.12460

**Published:** 2021-03-02

**Authors:** Wei Liu, Ahmad Beltagui, Songhe Ye

**Affiliations:** ^1^ Department of Engineering King's College London London UK; ^2^ Operations and Information Management Department Aston Business School Birmingham UK; ^3^ Institute for Manufacturing University of Cambridge Cambridge UK

## Abstract

As the COVID‐19 pandemic spread across the globe in the first quarter of 2020, demand for specialised equipment in hospitals soared. As a result, firms from a variety of sectors repurposed their design and manufacturing to create new products in days. By examining 80 cases of this accelerated innovation, the research investigates how a shared purpose drives change in the innovation process. It applies the lens of exaptation – the discovery of unintended functions for technologies – to explain how product complexity and ecosystem structure affect accelerated innovation in this context. The research extends the application of exaption to manufacturing as well as product design; it identifies a relationship between complexity, exaptation and ecosystems. The research suggests that the ability to exapt design and manufacturing can determine a firm’s ecosystem role. These results lead to implications for theory and for practice, during the response to and recovery from the crisis.

## 1 Introduction

The severity of the novel coronavirus and the speed with which it spread created unprecedented and unpredictable surges in demand for specific products such as hand sanitiser, personal protective equipment (PPE) and medical devices, notably ventilators. With quarantines, lockdowns and social distancing measures across the world restricting normal operations, many firms used available capacity to meet the demand through rapid repurposing of technologies. For example, on 15th March 2020, the UK government called on help from manufacturers to meet the National Health Service’s (NHS) predictions of demand for ventilators.^1^ Within 2 weeks, Dyson, a producer of household appliances such as vacuum cleaners, hand‐dryers and air‐purifiers announced it had designed and was preparing to produce 10,000 units of a new ventilator.^2^ A ventilator is a complex device that supports patients who are unable to breathe naturally. How can the creation of a functioning design be accelerated to this extent? And how have firms in a wide range of industries been able to repurpose their capabilities to do so? Answering these questions will help advance knowledge of innovation in general as well as guide responses to future crises.

Technological innovation normally follows a slow process of evolution over time. For example, the development of technologies from hammers (Basalla, 1989) to jet engines (Carignani et al., 2019) has been observed as a modification and refinement to adapt to specific functions. Each modification creates a new technology trajectory as incremental innovations help to improve performance and fit with the intended function. Alternatively, a new and unintended function may be found for a previously existing technology (Arthur, 2009). This is labelled as *exaptation* (Gould and Vrba, 1982), an ability to ‘pivot’ (Dooley and Som, 2018) from one function to another, without the need for a long and costly development process. This can take place through the repurposing of specific modules, for example, the magnetron, a radar component, was exapted to form the basis of the microwave oven (Andriani and Carignani, 2014). This process of exaptation may demand collaboration among specialised firms that work together for collective benefit, even where there may normally be competition between them. The result is an ecosystem (Iansiti and Levien, 2004), which offers the structure of a value proposition (Adner, 2017) and a set of niches that are filled by firms providing modules or services (Moore, 1993). Changes in the firms or technologies filling these niches cause the ecosystem to evolve (Beltagui et al., 2020). In the context of COVID‐19‐related repurposing, ecosystem formation and evolution can be seen in the collaborations between firms to design and produce innovations such as ventilators. Not only have these firms repurposed their production and developed or applied new technologies (Rapaccini et al., 2020), but also they have done so in collaboration with firms they may not previously have worked with (Chesbrough, 2020; Kuckertz et al., 2020).

The purpose of this research is to understand how repurposing has taken place in this context, to create knowledge for innovation practice in general and accelerated crisis response in particular. By analysing firms that have exapted their capabilities to create PPE (specifically face visors) and medical devices (ventilators), it develops the understanding of exaptation, ecosystem formation and evolution. The research demonstrates the interconnections between design complexity, manufacturing flexibility and ecosystem structure. The contributions of the research are threefold. First, it extends the exaptation concept to incorporate manufacturing processes as well as product architecture. Second it identifies a connection between complexity, exaptation and ecosystems – the more complex the product, the more likely that an ecosystem, rather than an individual firm – is involved in the exaptation. Finally, it proposes that the ability to exapt either products or manufacturing processes can determine the position a firm will take in an innovation ecosystem, and the opportunities available in future. The research suggests managerial implications that will help in the recovery from COVID‐19 as well as resilience and agility in future situations.

## 2 Background

### 2.1 Accelerated innovation

Innovation, the creation and commercialisation of new products, is essential to business and societal development. How to do it faster is a question that has vexed firms for decades (e.g. Gold, 1987). Answering this question is vital in the context of intense, globalised competition, where reducing development time (Griffin et al., 2019) or time‐to‐market (Pawar et al., 1994) can determine the profitability of first movers (Ellwood et al., 2017). Following Williamson (2016), we define accelerated innovation as *the capacity for dramatically faster and less costly development of products that are new to a firm*. It can be achieved through various methods, including teams working concurrently, rather than sequentially, frequent testing and iteration throughout the innovation or application of novel process technologies (Clark and Fujimoto, 1991; Brown and Eisenhardt, 1995). Flexible processes and organisation (Williamson, 2016), leadership experience and team tenure (Heirman and Clarysse, 2007) and the ability to elicit and rapidly respond to customer feedback (Williamson and Yin, 2014) have all been shown to contribute.

Despite the benefits that accelerated innovation can achieve, a number of studies have highlighted the trade‐offs, primarily the risk of poor quality (Bayus, 1997; Cankurtaran et al., 2013). Additionally, while Williamson and Yin (2014) point to successful examples of accelerated innovation in Chinese companies but suggest these rarely involve technological breakthroughs are not the focus of attention. In responding to the COVID‐19 pandemic, novelty of technologies was not always essential, whereas speed and quality are vital. For example, the suddenly identified need for ventilators required production of products meeting safety standards, to be rapidly scaled up. The existing literature on accelerated innovation focuses on internal processes, with inconclusive evidence regarding external collaborations (e.g. Heirman and Clarysse, 2007). Moreover, it considers either development of novel products, or modification of external designs (e.g. Williamson and Yin, 2014). Innovation in general, and in response to COVID‐19 in particular, can be accelerated by relying on external collaborations. For example, Chesbrough (2020) argues that open innovation, as well as repurposing existing manufacturing, are key, while Kuckertz et al. (2020) highlight reliance on relational capabilities as essential. These arguments lead to a suggestion that exaptation and ecosystems may play an important, but under‐investigated role in accelerated innovation.

### 2.2 Exaptation

Exaptation was originally proposed as an alternative evolutionary mechanism to adaptation, in biology (Gould and Vrba, 1982). While adaptation refers to features developed to achieve a particular function, exaptation refers to features developed for one function but later discovered to be for another. In an innovation context, exaptation is made possible because all of the possible future uses of a technology cannot be known in advance. A product can be exapted. For example, audio compact discs were exapted as a storage medium for computer data (Dew, 2007), while medicines to treat one condition are regularly exapted when other uses are identified for them. Alternatively, modules from a product architecture can be used as the basis of a new product. For example, lasers used in barcode scanners have been repurposed within surgical tools (Bonifati, 2010). The latter is referred to as modular exaptation, and can entail a change in the function of an artefact, or the function of one of its modules, or changes to both (Andriani and Carignani, 2014). Modular exaptation relies on the transfer of modules from one architecture to another, which may be referred to as *porting*, or *substituting* modules (Baldwin and Clark, 2000).

Exaptation may also be applied to process innovations and manufacturing capabilities. For example, Cattani (2005) examined the development of optical fibre. This was based on exaptation of Corning’s specialist glass manufacturing technology. Only when a need arose due to relevant developments in electronics and data transfer did the potential of the technology become clear. Exaptation, has therefore, has been achieved both through product design capability and manufacturing flexibility. Yet, it is rarely practiced by individual firms acting alone. Instead, it typically depends upon an innovation ecosystem, which brings together supply and demand, cooperation and competition (Ansari et al., 2016). For example, the success of Danish wind energy firms owes much to exaptation by an ecosystem of firms supported by government direction (Garud and Karnøe, 2003). Meanwhile the growth of the 3D printing ecosystem owes much to the entry of firms from a range of sectors (Beltagui et al., 2020). To understand how innovation as repurposing takes place, it is useful to understand each of these three elements – design capability, manufacturing flexibility and the ecosystems in which innovation takes place.

### 2.3 Design capability

Under conditions of high uncertainty and change, design is a particularly relevant driver of innovation (Auernhammer, 2020). Design capability can be defined as a collective capability held by an organisation that allows it to deploy a form of design (e.g. graphic design or mechanical design) to create or change products and services. Design is the ability to envisage and initiate changes to the world we live in (Evans et al., 1982). It is the application of human knowledge to the creation of artefacts – including physical products, and also intangibles such as services, software, contracts and rules – that perform specific functions (Simon, 1996; Baldwin and Clark, 2000). These artefacts can be considered as an integrated system of modules, each performing a function that contributes to the performance of the artefact. Complex technologies are artefacts that contain a large number of modules, with a correspondingly high number of interactions between them. Innovation can be seen as the process of designing the architecture, or redesigning the architecture, often by reusing modules from elsewhere (Henderson and Clark, 1990, Arthur, 2009). Baldwin and Clark (2000) envisage design in terms of actions designers use to manipulate the product modules, architecture or both. These modular design actions help to make a connection with exaptation, which can involve building artefacts using modules from another architecture (Andriani and Carignani, 2014).

### 2.4 Manufacturing flexibility

Design capability is not a requirement for manufacturing if open innovation (Chesbrough, 2020) means designs can be sourced externally from professional organisations (Berends et al., 2011) or other individuals (Liu et al., 2019). Producing these designs, however, requires manufacturing flexibility. Flexibility refers to the ability of a manufacturing system to respond quickly to changes in circumstances without excessive time, effort, cost or loss of performance (Beach et al., 2000). It can be a reactive capability to respond to adverse circumstances, but may also be used proactively to gain competitive advantage. For example, the modification of existing products, producing a wide mix of products or introducing new products are all valuable forms of flexibility (Malhotra and Mackelprang, 2012). Where demand is predictable and consistent, there is little need for flexibility. Instead, efficiency can be achieved by consistent production of large volumes and small varieties. Where firms operate in turbulent environments, however, flexibility can be achieved through the use of digital technologies such as 3D printing (Rong et al., 2020). Additionally, working in collaboration with other firms, in combination with digital technology can reduce product design and development time, in order to innovate faster (Cao and Dowlatshahi, 2005).

### 2.5 Innovation ecosystems

Innovation ecosystems are complex, adaptive systems (Choi et al., 2001) in which there is interaction and interdependence among firms operating across indeterminate boundaries. The term ecosystem can refer to the affiliation between firms that crosses industry boundaries (Moore, 1993), or to the structure around a common platform or value proposition, which firms contribute (Adner, 2017). An ecosystem incorporates both value creation and appropriation (Autio and Thomas, 2014). There is typically an ecosystem leader that plays a coordinating role, by shaping the vision (Liu and Rong, 2015) or offering a platform (Gawer and Cusumano, 2014) for other firms to contribute to. Iansiti and Levien (2004) distinguish between ecosystem leaders that act as dominators, extracting value from the firms in the ecosystem; and keystones, which help to create opportunities for others and profit by supporting the overall health and diversity of the ecosystem. Other firms occupy a particular niche, satisfying both their own and the ecosystem’s objectives (Nambisan and Baron, 2013; Dedehayir et al., 2018). As with biological ecosystems, these niches may be occupied by new entrants into an ecosystem, whose specialised capabilities may be useful for fulfilling required functions. Innovation ecosystems typically evolve over a long‐time period, due to the entry of new firms, who compete for niches, leading to eventual exits (Moore, 1993). Exaptation can play an important role in the formation of ecosystems, and also in their evolution, as firms bring their specialised capabilities into a growing ecosystem (Beltagui et al., 2020). In the context of COVID‐19, this formation may have happened very rapidly, due to necessity. Firms entered into the newly forming ecosystem by repurposing, that is, changing the function to which products were applied. In the new ecosystem, diverse firms are united by a new and shared purpose: to reduce and reverse the spread of COVID‐19, as opposed to marketing their particular products or services. Given that the literature on accelerated innovation (e.g. Williamson, 2016; Elwood et al., 2017) says little about ecosystems, we investigate how these ecosystems formed, which specialised capabilities were exapted, and whether ecosystems may be connected with accelerated innovation.

**Figure 1:**
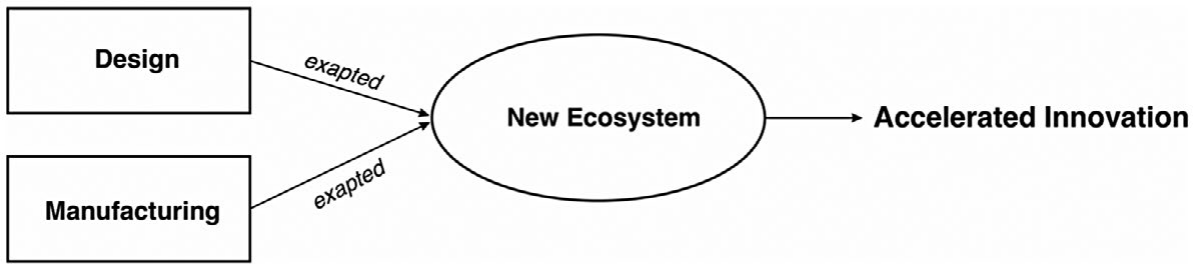
A theoretical framework of this research.

Figure 1 shows the theoretical framework of this research, demonstrating the relationship among the key concepts reviewed above.

## 3 Methods

To understand the role of exaptation in responding to COVID‐19, multiple cases of firms involved in repurposed production were examined. Multiple case studies are regarded as a useful way to build theory (Eisenhardt, 1989) and recent studies have increasingly relied on secondary data to develop them. Given the availability of easily accessible secondary data, the internet has, therefore, become a viable alternative to traditional data collection when conducting qualitative studies (Kozinets, 2002). For example, Franzoni and Sauermann (2014) use examples of online projects to contribute an understanding of crowd science and its role in innovation. They build an understanding of each crowd science project by constructing narratives using secondary data.

A list of 91 UK‐based organisations that contributed to production of PPE and equipment for NHS was identified through media reports and trade publications.^3^ These are mostly manufacturing firms from a variety of industries and sectors, and also included research institutes who contributed primarily to the design and development of products. We consider them to be cases of innovation if they created or contributed to the creation of products that were new to the organisation. They represent accelerated innovation because the list of firms was compiled a few weeks after the pandemic was declared – a very short timescale for new products to be launched. And they represent exaptation where they used product modules or manufacturing for a purpose that differed from the norm. After removing cases that did not meet these criteria, 80 cases remained. These are considered by product – face visors; ventilators; gowns, aprons and scrubs; and hand sanitiser. We examine why some of these were produced by firms working independently, while others were delivered by multiple firms that formed or joined ecosystems to deliver a collaborative response.

Data were collected by examining the official websites, social media accounts and press releases of each of the identified organisations. This was supported by examination of publicly available documents such as company registration, industry sector and available accounts.

Data analysis involved coding each cases, by design capabilities and manufacturing flexibility. This allowed the basis for exaptation (of products and processes) to be understood. The ecosystem dimension was examined by considering the trajectory taken, for example, from producing beverages for consumers and hospitality sectors to hand sanitiser for medical use. This represents a move from one ecosystem into another. Additionally, the role taken within the new ecosystem was coded, following Iansiti and Levien’s (2004) framework. The next section provides an overview of the analysis and findings from examining the repurposing activity in these organisations.

## 4 Findings

A number of patterns can be identified by analysing the repurposing undertaken in each case.

Table 1 provides an overview of the most common products created through repurposing, including what the organisation previously focused on, and the complexity of the resulting innovation. For example, the most commonly produced face‐shields may consist of three components – a headband, a transparent shield and a clip to hold the shape. This is an architecture with few modules and can be considered low in complexity, compared with a ventilator consisting of several interdependent mechanical and electronic modules. Similarly, the manufacturing technologies typically used, give some indication as to the ease of manufacturing. For example, surgical scrubs can be produced by sewing, whereas hand sanitiser requires both bottle production (normally blow‐moulding of plastic) as well as chemical processing.

**Table 1:** Overview of the main products produced through repurposing in response to COVID‐19

Products	Number of modules	Product design complexity	Materials	Manufacturing technologies	Medical regulation requirements	Ecosystem complexity
Face‐shield/visor	3–5	Low	Polycarbonate/cellulose acetate sheets, elastic foam strap, mylar, etc.	3D printing/extrusion/injection moulding, etc.	Low	Low
Surgical facemask	3–7	Low	Polypropylene fibres or cloth	Melt blowing, ethylene oxide sterilisation, spot welding, sewing, etc.	Intermediate	Low
Gloves	1	Low	Latex, nitrile rubber, polyvinyl chloride and neoprene	Dispersion, coagulant, vulcanising, etc.	Intermediate	Low
Hand sanitisers	3–6	Low	Ethanol/isopropyl alcohol, purificated water, gel, etc.	Blow‐moulding, batch mixing, purification, filling, etc.	Intermediate	Intermediate
Gown/apron/scrubs	1–5	Intermediate	Polythene, etc.	Cutting sewing ironing, etc.	Intermediate	Intermediate
Ventilators	>10	High	Various materials and components from metal, rubber, plastics, electronics, etc.	Casting, CNC, laser cutting, waterjet cutting, 3D printing, injection moulding, etc.	High	High

The table also evaluates the ecosystem complexity – whereas face visors can be produced by individuals in possession of a desktop 3D printer, ventilator design is much more complex and demands close collaboration between multiple actors. For instance, a number of firms collaborated to reverse engineer and produce a Continuous Positive Airway Pressure (CPAP) device, a form of ventilator. The firms included Martin’s Rubber Company, which typically produces rubber products for defence and automotive applications, but created components for the ventilator breathing tube. Similarly, Spirax Sarco, a producer of steam‐related products for process industries such as beverages and oil production, developed a connector to manage oxygen flow. Each of these firms repurposed their products, their manufacturing or both. Additionally, in order to accelerate their innovation effort they joined a newly formed ecosystem, led by University College London and Mercedes AMG, and spanning industry boundaries.

### 4.1 Repurposing design

Appendix A presents a full tabulation of the cases explored in this study in terms of repurposing to ventilators, face‐shields/visors, hand sanitisers and gowns/aprons/scrubs. The cases under investigation include some examples where no new design was required since the repurposing does not require any change to a product architecture or modules. For example, firms producing products that could be used without modification, focus on scaling up their production. In several cases, however, products are redesigned. This is especially true in the development of ventilators. Using the example of Spirax Sarco mentioned previously, design capability is required to create a module that processes oxygen, by repurposing products that process steam. Given the specialised nature of the task, the firm requires a base of design capability, from which to create the component. The challenge of designing a ventilator can be seen in the fact that several consortia tried but failed to produce a working design, or to gain certification. In particular, specifications changed rapidly as understanding of the disease and how to treat it became clearer. Rapid design decisions, made in order to quickly go into production, made some products unsuitable once the specifications were updated. And the lead time for adequate testing means production of new products, no matter how quickly they are designed, may be slowed.

In contrast, for less complex products, creating the artefact involves lower levels of uncertainty and requires a lower level of capability. This can be seen in the wide range of organisations creating and assembling face‐shields as the list of firms and their starting sectors in Appendix A suggest. The design is relatively simple, but more importantly, a number of open‐source designs were developed and widely shared. This means that new design effort is not required – anyone with a desktop 3D printer can download and produce face‐shields to meet the local demand for PPE (Chesbrough, 2020). In a crisis scenario, the normal development processes, with multiple design cycles are not feasible. Instead, firms collaborated to quickly develop designs by reverse engineering existing products and repurposing their own products to create the modules. Alternatively, they made use of existing designs that were shared by existing manufacturers (in the case of ventilators) or made openly available for download (face‐shields).

At least 30 organisations have started producing face‐shields. They come from sectors including education, precision engineering, aerospace and defence, medical devices, plastic packaging and others. For example, TRB Lightweight Structures, an engineering firm used an open‐source design from Foster + Partners. Amtico, a flooring manufacturer, modified the Luxury Vinyl Tiles they produce in order to create headbands for face‐shields. Meanwhile, Jaguar Land Rover, an automobile manufacturer, with in‐house design expertise, produced its own design, which has been made available to additive manufacturers and suppliers. Similarly, Kite Packaging and ICL Tech, contract manufacturers of packaging and plastics, produced their own designs. In these latter cases, design capability was used.

For ventilators, 35 organisations were identified as repurposing, from eight sectors including research and education, precision design & engineering, aerospace and defence, automobile, chemicals, plastics and electrical equipment. More firms are involved, but from fewer sectors than for face‐shields, and none of these firms acts alone in designing and producing ventilators. Dyson designed and developed a new ventilator named ‘CoVent’ using existing design modules from its vacuum products. It collaborated with other firms including JCB – an earthmoving equipment producer that contributes sheet metal housings for the new product. On the contrary, BAE Systems, a large defence contractor, which typically works on complex technology projects, was able to design an entirely new ventilator named ‘AirCare ventilator’ within 3 weeks with its in‐house design and engineering teams.

### 4.2 Repurposing manufacturing

While design capability enables the repurposing of modules to create new product architectures, the ability to make these modules is crucial to accelerating innovation. Two approaches can be seen in the firms that were able to repurpose their manufacturing capability – technology‐driven flexibility and specialisation.

Using the open‐source designs described previously, the flexibility afforded by 3D printers is very valuable. 3D printers are digital fabrication tools that build objects directly from computer models and are, therefore, capable of producing almost anything. They are widely used by individuals and firms for rapid prototyping and product development, which makes them suited to rapid response to a crisis. Firms using them to make face‐shields include Scales & Models, an architectural model maker, and Mondelēz, a producer of confectionery which repurposed the 3D printing facilities it normally uses to make chocolate sculptures at its Cadbury Bournville site. Similarly, contract manufacturers that operate technologies such as Computer Numerically Controlled (CNC) machines, were able to repurpose their production lines to accommodate ventilator parts, for example, AE Aerospace. In these cases, the manufacturer regularly manufacturers parts to customers specifications, and is able to deliver even where the parts are unfamiliar.

Specialisation was also observed, where complex products requiring an ecosystem approach, involved firms repurposing using specialised manufacturing facilities to create specific modules with specific requirements, in a short space of time. For example, firms that operate injection moulding equipment, including What More UK, a producer of plastic homeware goods and Sabre Plastics Tooling, were able to produce parts for face‐shields and ventilators, respectively, in higher volumes. Beyond these products, the shortage of other PPE, as shown in Appendix A, was mostly addressed by apparel producers, with the capability and capacity to produce them. For example, Wearwell, a producer of workwear for safety, repurposed its production lines to make workwear for medical personnel, including some products treated with a proprietary antiviral agent. Similarly, clothing and textile production lines at Barbour and John Lewis, respectively, were repurposed to PPE. In some cases the extent of repurposing was greater, notably Where The Trade Buys, a printing firm, which used its capability to print promotional material on plastics to create PVC aprons customised with text or graphics, for frontline workers.

Production of hand sanitiser largely follows the formulation recommended by the World Health Organisation (WHO, 2010). Flexibility can be seen in the ability of firms in beverages and chemicals sectors to repurpose their production, for example, Alderman’s Drinks using its experience of handling high strength alcohol and INEOS, a producer of ethanol and isopropyl alcohol, which devoted production lines to hand sanitiser. While these firms have the capability to process chemicals, they may lack the capability to produce suitable bottles, unlike Marches Bottling and Packaging, which together with its sister cider company of Celtic Marches Beverages could deliver both modules.

### 4.3 Repurposing ecosystems

To rapidly increase the production of ventilators, a consortium named VentilatorChallengeUK was created. This represents an ecosystem with members from across industries including aerospace, automotive and medical sectors, along with government and academic backing. The members with strong experience in producing ventilators led the challenge, and others brought expertise in precision manufacturing and engineering to support the ecosystem. This new ecosystem largely formed by repurposing firms' existing design capability and manufacturing flexibility. This repurposing has the potential to speed up the innovation process, but it relies on a shared purpose for the ecosystem to form around. The chair of the consortium, Sir Dick Elsy stated, *harnessing the manufacturing muscle of big companies to increase the output of specialist firms had proved to be the right approach*.^4^ One of the typical examples is increasing ventilator production of Smiths Medical, a member of the consortium to meet the predicted demand created by COVID‐19.

To rapidly scale up production of its ‘paraPAC plus’ ventilator, Smiths Medical was supported by firms with no track‐record of producing medical parts or products. For example, AE Aerospace, switched its low‐volume aerospace production lines to make over 6,000 specific complex milled parts for the ‘paraPAC plus’ ventilator within 2 weeks from receiving a design drawing. BAE Systems, a defence company, supported ramp up production and provided integrated, tested sub‐systems and components for the ventilators. Produmax, a precision engineering company, repurposed its lathes to produce brass, steel and plastic components of air flow control units of Smiths’ ventilators. These new participants in the ventilator ecosystem prompted the production increase of Smiths Medical at Luton to four‐times the usual amount through releasing their manufacturing capabilities from the current business to the ventilator business. In the meanwhile, Smiths Medical also provided intellectual property and technical advice to other firms to produce the ‘paraPAC plus’ ventilator.

Unlike AE Aerospace or Produmax, Nissan, the automobile manufacturer, became a kind of ‘logistics centre’, processing modules sent in from volunteers across the United Kingdom who used their own 3D printers to make visor parts, such as elastic headband, frame and see‐through visor.

Nissan established the parts processing line at the Sunderland factory to pack and distribute the ready‐to‐assemble visors to the NHS, the format of which can minimise the damage risk during transport and maximum volume can be delivered at once. In addition to providing logistics, Nissan also funded the tooling for injection moulding – supporting the ecosystem by helping to increase production volumes.

## 5 Discussion

**Figure 2:**
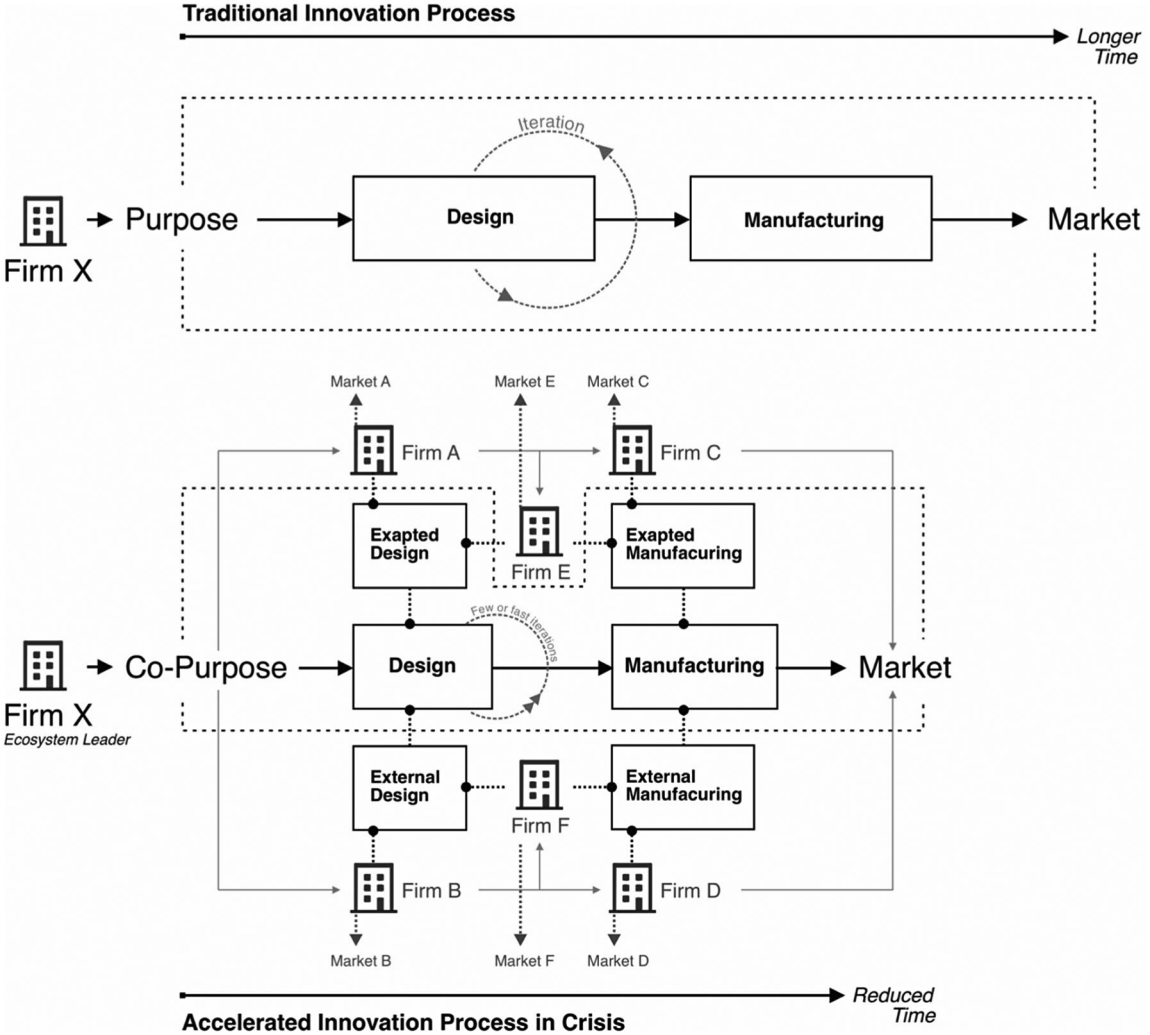
A proposed process for accelerating innovation through exaptation.

The cases under investigation illustrate how accelerated innovation takes place. In this crisis, affecting all countries and industries, ecosystems established a common purpose. According to Liu and Rong (2015) ecosystem leaders develop a co‐purpose, in this case meeting demand for medical equipment to combat a pandemic, which encourages other firms to join the ecosystem and innovate. In a time of crisis, we argue that leading firms in the ecosystem share common purposes (co‐purpose), for example, the development of products on urgent demand, with other ecosystem firms to encourage them to develop products together, which is echoed by Liu and Rong (2015). Firms may either use exapted designs from existing modules or architectures, or external designs created by other firms to supply their design development. The design iterations would be fewer in number or faster in speed. In the meanwhile, firms may repurpose their production lines to manufacture new products or collaborate with external manufacturers to complement their own production inadequacy or increase productivity. In line with their common innovation objectives, firms in either inside or outside the ecosystems work together to accelerate the innovation process by repurposing existing technology, rather than developing new technology, as shown by the reduced timescales indicated in Figure 2.

**Figure 3:**
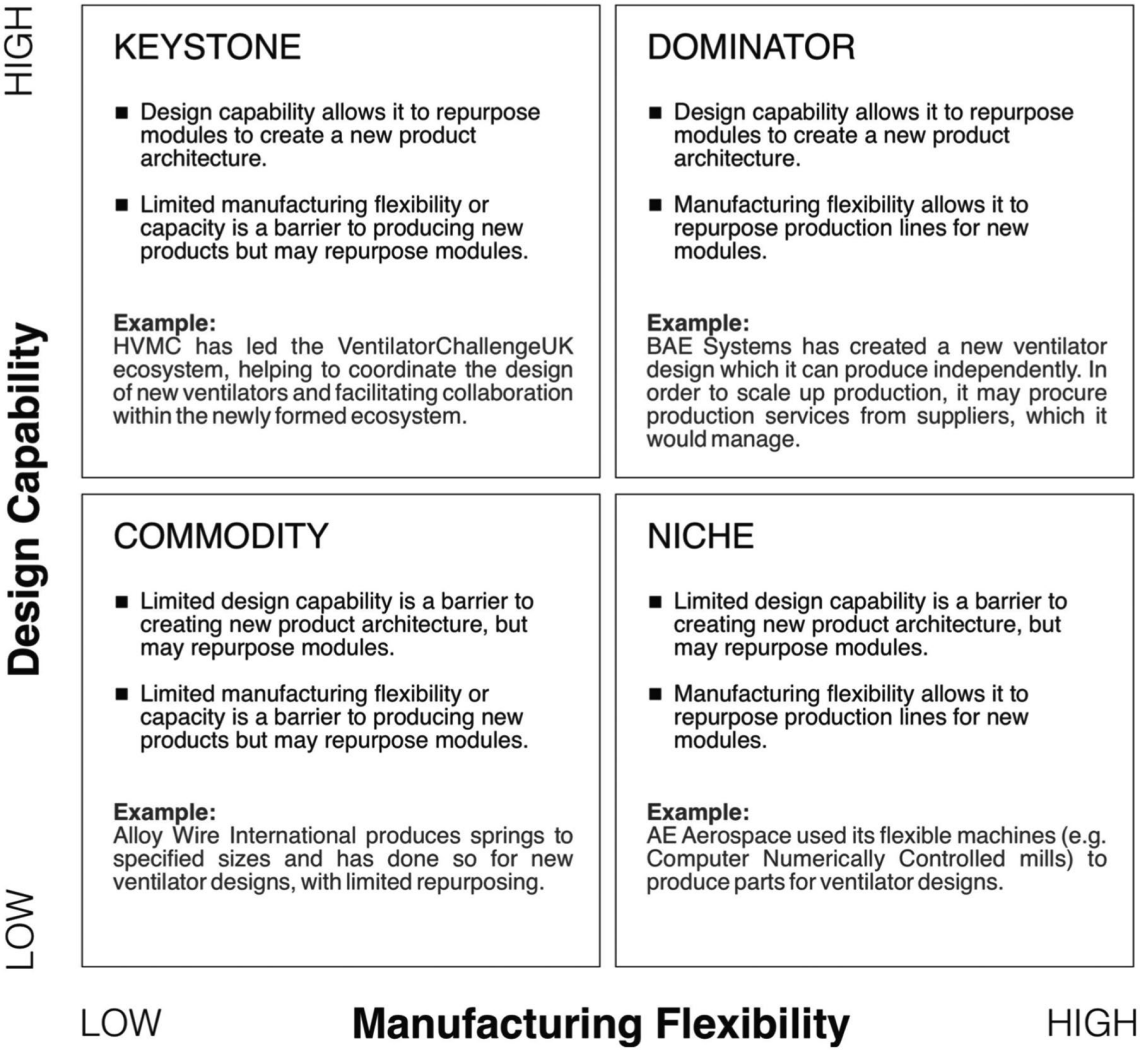
Ecosystem roles during exaptation – based on Iansiti and Levien (2004).

This research set out to understand the acceleration of innovation in response to the COVID‐19 crisis. By examining design capability and manufacturing flexibility, through the lens of exaptation, it produced findings connecting product architecture to ecosystem structure. In particular, the connections between complexity, the ability to repurpose product designs and manufacturing are related to ecosystem roles in Figure 3. It draws on Iansiti and Levien’s (2004) distinction between keystones (which seek mutual benefit for the whole ecosystem) and dominators (which seek to manage or control the ecosystem) and between niche players and commodities. All of these have been crucial to the accelerated innovation in this context but it may be interesting to note that the roles are not solely linked to their size or previous position. Instead, the ability to repurpose products through design capability and production lines through manufacturing flexibility help them to take a leading role. While the COVID‐19 response is expected to be temporary, the innovations and collaborations that have been achieved may lead to ongoing opportunities. A case in point is Marches’ aim to make hand sanitiser production and bottling a permanent, profit‐making line.^5^

### 5.1 Theoretical contributions

The contributions of this research to theory are threefold. First, it extends the application of exaptation, showing how it applies both to design and to manufacturing processes. Previous literature has examined the phenomena through the transfer of technologies from one application to another (e.g. Garud et al., 2018) or the transfer and repurposing of product modules (Andriani and Carignani, 2014). Alternatively, at a firm level, the already existing capability to manufacture something new, has been considered (Cattani, 2005). By including both product and process technologies in the equation, the cases investigated demonstrate that both can support exaptation, which in turn can support accelerated innovation.

Second, the research builds on the connections between exaptation and ecosystems that are either implicitly (e.g. Garud and Karnøe, 2003) or explicitly (Beltagui et al., 2020) expressed in the literature. A relationship is identified between ecosystems and complexity. As complexity – of products and manufacturing processes – increases, it appears that the need for ecosystems is also higher. Ecosystems are particularly useful for accelerating innovation because they enable the exchange of capabilities across typical industrial boundaries. Examples of collaborations between traditionally unrelated firms facilitate exaptation as modules are identified to fulfil a purpose, at the same time resulting in an ecosystem niche being filled (Moore, 1993).

The third contribution is the proposition that design capability and manufacturing flexibility are determinants of a firm’s ecosystem role. In this research, design capability is observed in those firms that lead the newly formed ecosystems, since they design the architecture of the new products created. Meanwhile manufacturing flexibility is required in the ecosystem and may be provided by important niche actors, or by firms leading and controlling the ecosystem. The research builds on Iansiti and Levien’s (2004) categories, but applies them to the formation of ecosystems, something that is rarely investigated in the innovation literature.

The proposition suggests implications and directions for both practice and further research.

### 5.2 Managerial Implications

For practitioners involved in managing innovation, the research offers a number of suggestions and recommendations. Exaptation, the repurposing of product and process technologies, may be seen as a fruitful means of accelerating innovation. It is observed in this context of crisis response, but may also be applicable to economic recovery. It has the benefit of speeding up development through the reuse of existing modules, in creative combinations and helps explain how firms can develop complex products for the first time, in a matter of days or weeks not months and years. To support exaptation, working in collaboration with an ecosystem of diverse partners is important. Collaboration in this case is guided by political encouragement and the threat of an existential crisis. Achieving the same collaboration in future may be more challenging, but worth pursuing. And assuming such ecosystems can be created, managers should consider the role their firm should play in them. The research identifies the importance of design capability to leading ecosystems and manufacturing flexibility in playing an active role. Both of these should therefore be cultivated, to improve innovation performance as well as preparedness for future crises.

Meanwhile, the risks of innovating too quickly should be considered. COVID‐19 presented an almost entirely unprecedented situation, in which not only the size and location, but also the nature of demand changed rapidly. For ecosystems formed to deliver ventilators, this was problematic due to specifications changing as the most effective treatments were identified, sometimes leaving their efforts in vain as orders were cancelled. Even in these circumstances, however, the ability to exapt can soften the blow. While the products they developed may have been ultimately unsuitable, or not approved due to insufficient testing, the resulting products, processes and partnerships can be put to use in future, if and when another common purpose can be found.

### 5.3 Limitations and future work

While the research highlights the benefits of accelerated innovation, there are limitations and some of these apply to the research itself. In order to document ongoing phenomena, the research relies on secondary data available in the public domain. These accounts are in general verified from multiple sources, but present a limited perspective. To further develop the findings, detailed in‐depth case studies should document what has happened along with the successes, failures and best practices. A related limitation is the sample of firms investigated. The research does not claim to be comprehensive in capturing all of the repurposing activities that have taken place in the United Kingdom, let alone internationally. Further research might take a broader perspective, for example, through survey research to take stock of the extent of repurposing and seek to explain the mechanisms by which exaptation takes place. This crisis is unique in its global nature, affecting every nation and every economic sector and it, therefore, provides a unique opportunity to understand exaptation. This may be achieved through a study of an industry, for example, how the cosmetic sector has responded by repurposing to create hand sanitiser. Finally, the risk of studying phenomena as events unfold is that the end of the story is unclear. For example, where ventilators were developed but did not meet the changed specifications or could not be tested in time, there may be a future opportunity to pursue. Whether firms from aerospace, automotive, consumer goods and other sectors build on the outcomes they have achieved, to develop their reach in the healthcare market remains to be seen. Longitudinal research that helps to explain what will happen next could be vital in understanding the long‐term effects of accelerated innovation through exaptation.

## 6 Conclusion

The COVID‐19 crisis is unprecedented in modern times in terms of the spread of infection and severity of impact. With the demand for vital equipment soaring beyond normal production or safety stock, accelerating the development and delivery of equipment was essential. While lessons can be learned in relation to preparedness for future crises, the experience may also be beneficial for ‘normal’ economic activity. Both design capability and manufacturing flexibility are valuable for innovation in conditions of market or technological turbulence (Candi and Beltagui, 2019; Auernhammer, 2020). And this research suggests that they are both enablers of accelerated innovation. The COVID‐19 pandemic created a need for repurposing due to the very specific needs (e.g. ventilators over any other medical device). Achieving this level of focus may be difficult outside of the unique life and death nature of the current context. Yet, if harnessed, product development lead times may be reduced, to support recovery from the crisis.

## Notes

^1^

http://news.bbc.co.uk/1/shared/bsp/hi/pdfs/15032001.pdf , last accessed 21 December 2020.

^2^

https://edition.cnn.com/2020/03/26/tech/dyson‐ventilators‐coronavirus/index.html , last accessed 21 December 2020.

^3^

https://www.themanufacturer.com/articles/every‐uk‐manufacturers‐helping‐to‐produce‐ppe‐and‐equipmentfor‐nhs‐workers/ , last accessed 14th May, 2020.

^4^

https://www.theguardian.com/business/2020/apr/21/we‐made‐right‐choice‐in‐ventilator‐race‐says‐ukconsortium‐head , last accessed 14th May 2020.

^5^

https://hwchamber.co.uk/marches‐bottling‐and‐packaging‐ltd‐team‐up‐to‐produce‐hand‐sanitiser/ , last accessed 15th May 2020.

## Appendix A. Full tabulation of case firms producing face‐shields, ventilators, hand sanitiser and gowns/aprons/scrubs

**Table:**

Firms	Industry	Before exaptation	After exaptation
*Repurposing to produce ventilators*			
Manufacturing Technology Centre	Research & education	Manufacturing processes, technologies and system solutions	Scalable new intubation shield for use with ventilators
Alloy Wire International	Precision design & engineering	Precision drawn round wire, flat wire, profile wire, bars and wire rope in nickel alloys	Ventilator components for the VentilatorChallengeUK consortium
A&M Edm		EDM wire and spark erosion; precision engineering solutions	Ventilator components for the VentilatorChallengeUK consortium
Beverston Engineering		Precision engineering and services, 5‐ axis machining	Ventilator components
CNF Precision Engineering		Precision machining and services, CNC machined components	Ventilator components
Envisage Group		Engineering service provider, bespoke engineered vehicles and products	A new portable ventilator design
European Springs & Pressings		Spring manufacturing and high speed press technology	Mouthpiece springs for ventilators
H. V. Wooding		Precision engineering and services, wire erosion, busbars, motor lamination, presswork & electroplating	Ventilator components for the VentilatorChallengeUK consortium
JJ Churchill		Precision machining and treatment processes	Ventilator components for the VentilatorChallengeUK consortium
Martin’s Rubber		Precision elastomeric products and rubber engineering	Silicone ‘flag’ for the breathing tube of the UCL‐Ventura breathing aid
Nasmyth Group		Precision engineering solutions and mental treatments	Ventilator components
Precision Micro		Photochemical etching service for precision metal components	Ventilator components
Produmax		Precision engineering and services, flight control components	Ventilator components for air flow control units of Penlon and Smiths Medical ventilators
Protolabs		Low‐volume 3D printed, CNC‐machined, sheet metal and injection‐moulded custom parts	Ventilator masks
Renishaw		Measurement, motion control, spectroscopy and precision machining	Ventilator components for the VentilatorChallengeUK consortium
SL Engineering		Rigid tube assemblies and precision machined components	Ventilator components for the VentilatorChallengeUK consortium
Spirax Sarco		Steam management systems and peristaltic pumps and associated fluid path technologies	Quick‐fit connectors managing manage the flow of oxygen for the UCL‐Ventura breathing aid
Amphenol Invotec	Electrical equipment, appliances & machinery	Complex PCBs	PCBs for ventilators
Citizen Machinery UK		CNC Lathes machines, bar automatics	Turn‐milled components for ventilators
C R Clarke		Thermoforming and plastic fabrication equipment	A new Continuous Positive Airway Pressure (CPAP) ventilator
Dyson		Household appliances	A new CoVent ventilator
JCB		Equipment for construction, agriculture, waste handling and demolition	Steel housings for the new design of ventilator from Dyson
Plexus		Electronic components such as antennas, switches and waveguides	Production of the new design of ventilator from Babcock
PP Control & Automation		Electrical and electronic control systems and assemblies, cable harnesses	Harnesses for the VentilatorChallengeUK consortium
AE Aerospace	Aerospace & defence	Fabrication of aerospace metalwork and components	Complex turned/milled parts for the Smiths ‘paraPAC plus’ ventilator
BAE Systems		Civil and military aerospace; defence electronics; naval vessels; munitions; land warfare systems	A new AirCare ventilator; and Ventilator components for the VentilatorChallengeUK consortium
Babcock		Complex engineering services for national defence	A new Zephyr Plus ventilator
Hyde Aero		Aerospace assemblies and components	Ventilator components for the VentilatorChallengeUK consortium
Rockford		Wiring, interconnect & system solutions	Wiring assemblies for ventilators
Brandon Medical	Medical device & equipment	Medical lighting, power & control systems, architectural products and medical audio‐visual systems	Oxygen probes for ventilators
INOVUS Medical		Surgical and medical simulation equipment	Ventilator components
McLaren Group	Automobile	Sports and super cars, motorsport and technology	Ventilator components for the VentilatorChallengeUK consortium
Röchling	Plastics	Thermoplastics and composites for technical applications	Ventilator components for the VentilatorChallengeUK consortium
Sabre Plastics Tooling		Ultrasonic plastic welding tooling, horns, fixtures and jigs	Ventilator components
Ultraseal International	Chemicals	Porosity sealing in cast, sintered and electronic components	Ventilator components
*Repurposing to produce face shields*			
Advanced Manufacturing Research Centre	Research & education	Practical research into advanced machining, manufacturing and materials	Face shields
Axiom GB	Electrical equipment, appliances & machinery	Sortation systems, conveyors and mechanical handling systems	Plastic parts for face shields
Composite Integration		Resin transfer moulding and resin infusion	Face shields
Dextra Group		Electric lighting equipment	Visor headsets
Numatic International		Commercial and industrial cleaning and maintenance equipment	Face shields
Oxley Group		LED lighting, EMI filters and electronic components	Face shields
Parafix		Bespoke adhesive components	Face shields
Rototherm Group		Industrial instrumentation and services	Face shields
CHX Products	Plastics	Plastic promotion products	Face shields
DisplayMode		Point‐of‐sale displays and retail materials	Face shields
ICL Tech Ltd		Plastics thermoforming and fabrication	Face shields
Luminati		Retail display solutions	Face shields
Incontrast – STI Line Ltd		Sales promotional displays	Face shields
What more UK		Plastic storage and homeware products	Face shields
Kite Packaging	Packaging	Corrugated cardboard boxes, bubble wrap, polythene and cardboard packaging	Face shields
LJA Miers		Conversion and lamination of plastic, rubber, foam and fabrics	Face shields
A&M EDM	Precision design & engineering	EDM Wire and spark erosion; Precision Engineering solutions	Visors and chin pieces for face shields
Blackman & White		CNC machines versatile; Cutting machines	Face shields
TRB Lightweight Structures		Advanced and lightweight composite products	Face shields
The Royal Mint		Striking of coins	Face shields
Scales & Models		Sculpture, model making, prototyping and general 3D art	Face shields
INOVUS Medical	Medical device & equipment	Surgical and medical simulation equipment	Face shields
Surgical Dynamics Ltd		Surgical equipment and laparoscopic instruments	Face shields
Amtico	Building & materials	Flooring	Visor headbands
BLOC Blinds		Blinds	Face shields
BAE Systems	Aerospace & defence	Civil and military aerospace; defence electronics; naval vessels; munitions; land warfare systems	A newly designed face shield
Mondelēz International	Food	Cookie, chocolate and cracker	Visor headbands
Where the Trade Buys	Printing	Printing and graphic design	Face shields
Jaguar Land Rover	Automobile	Motor vehicles	A newly designed face shield (share design files using a royalty free licence)
Nissan		Motor vehicles	Visor assembly and delivery
*Repurposing to produce hand sanitiser*			
Alderman’s Drinks	Beverage	Arlu Rum; Didsbury Gin	Hand sanitiser
Manchester Gin Distillery		Gin	Hand sanitiser
HMG Paints	Chemicals	Paints and coatings	Hand sanitiser
INEOS		Petrochemicals	Hand sanitiser
PV3 Technologies		Electrochemical materials	Hand sanitiser
Marches Bottling and Packaging	Packaging	Bottling, kegging, cans and boxing	Bottles for hand sanitiser
*Repurposing to produce gowns/aprons/scrubs*			
Barbour	Apparel & fashion	Clothing, footwear and accessories	Gowns
Burberry		Clothing, footwear and accessories	Gowns
Cookson & Clegg		Community clothing	Scrubs
David Nieper		Women's apparel	Scrubs
Oliver Harvey Chef Wear		Chef whites, restaurant aprons and denim aprons	Aprons
Ventura Clothing		Clothing and apparel sourcing solutions	Gowns
Wearwell		Workwear for safety	Scrubs
John Lewis	Retail	Home and garden furnishings, electricals and fashion	Gowns
Laura Ashley		Home furnishings and fashion	Scrubs
Where the Trade Buys	Printing	Printing and graphic design	PVC Aprons
Bio‐Pack	Packaging	Compostable Packaging Solutions	Plastic Aprons
